# Effect of Cerebral Small Vessel Disease Burden on Outcomes in Patients With Acute Ischemic Stroke Receiving Endovascular Treatment

**DOI:** 10.3389/fnagi.2022.800617

**Published:** 2022-06-13

**Authors:** Hao Huang, Weifeng Zong, Xu Tong, Xue Tian, Anxin Wang, Baixue Jia, Jing Zhao, Lingshan Wu, Xirui Zhou, Yinping Guo, Yi Zhang, Zhiyuan Yu, Yilong Wang, Yongjun Wang, Xiang Luo, Zhongrong Miao

**Affiliations:** ^1^Department of Neurology, Tongji Hospital, Tongji Medical College, Huazhong University of Science and Technology, Wuhan, China; ^2^Department of Interventional Neurology, Beijing Tiantan Hospital, Capital Medical University, Beijing, China; ^3^Department of Epidemiology and Health Statistics, School of Public Health, Capital Medical University, Beijing, China; ^4^Beijing Municipal Key Laboratory of Clinical Epidemiology, Beijing, China; ^5^China National Clinical Research Center for Neurological Diseases, Beijing, China; ^6^Department of Neurology, Beijing Tiantan Hospital, Capital Medical University, Beijing, China

**Keywords:** cerebral small vessel disease, MRI, ischemic stroke, endovascular treatment, outcome

## Abstract

**Background:**

Cerebral small vessel disease (SVD) is common in the aging population. The study aimed to evaluate the effect of SVD on functional outcomes in patients with acute ischemic stroke (AIS) receiving endovascular treatment (EVT).

**Methods:**

From a prospective registry, we selected patients with AIS receiving EVT. SVD features, including white matter hyperintensities (WMH), lacunes and brain atrophy, were assessed on MRI and a validated SVD score was calculated to reflect the total SVD burden.

**Results:**

Among 137 patients included, 106 had none-mild SVD burden and 31 had moderate-severe SVD burden. The moderate-severe SVD burden group showed a significantly higher modified Rankin Scale score at 90 d (median, 4 versus 1 points, adjusted common odds ratio 0.32 [95% CI, 0.14–0.69], *P* < 0.01) and a significantly smaller improvement of NIHSS at 24 h (median, –3 versus –3 points, adjusted β coefficient 4.02 [95% CI, 0.57–7.48], *P* = 0.02) and 7 days (median, –4 versus –6 points, adjusted β coefficient 4.71 [95% CI, 1.06–8.36], *P* = 0.01) than the none-mild group. There was no significant difference in successful recanalization, death within 90 days, symptomatic intracranial hemorrhage within 24 h between two groups (all *P* > 0.05). Additionally, for each single SVD feature, brain atrophy and WMH, but not lacunes, were associated with the functional outcome.

**Conclusion:**

Moderate-severe SVD burden was associated with poor early and late functional outcomes in patients with AIS receiving EVT. Our results suggest that SVD score may act as a good predictor of outcomes in these patients.

## Introduction

Cerebral small vessel disease (SVD) is a group of pathological processes affecting the small vessels of the brain and associated with an increased risk of cognitive impairment and stroke (Pantoni, 2010). Although alterations in small vessels are not detectable with current *in vivo* neuroimaging methods, the SVD-related brain parenchymal lesions, including white matter hyperintensities (WMH), lacunes, cerebral microbleeds, enlarged perivascular spaces and brain atrophy, can be observed with neuroimaging and identified as image markers of SVD (Wardlaw et al., 2013a).

Endovascular treatment (EVT) is an effective procedure for treating patients with acute ischemic stroke (AIS) with large vessel occlusion (LVO) (Goyal et al., 2016). Previous studies showed that the outcome in patients with AIS receiving intravenous thrombolysis (IVT) was associated with preexisting SVD, including WMH (Charidimou et al., 2016), lacunes (Huo et al., 2019), and cerebral microbleeds (Charidimou et al., 2017). The association between SVD and the outcome in patients receiving EVT has also been investigated. Among these SVD markers, brain atrophy was reported to be associated with outcome and mortality after mechanical thrombectomy (Diprose et al., 2019; Lauksio et al., 2021), whereas cerebral microbleeds not (Shi et al., 2016; Derraz et al., 2021). The association between WMH and clinical outcomes has been reported with conflicting results (Atchaneeyasakul et al., 2017; Boulouis et al., 2019). Most previous studies have focused on single markers, but it remains unknown whether the combination of these markers could affect the outcome in patients receiving EVT.

To fully evaluate the effect of SVD on the brain, a combined assessment of these image markers, rather than a single marker, is preferred. Recently, an SVD score consisting of three SVD markers (WMH, lacunes, and atrophy) was proposed to assess the overall burden of SVD and applicable to the evaluation of both computed tomography (CT) and magnetic resonance imaging (MRI) scans (Arba et al., 2017b,^2019^). One study showed that, when assessed with CT scan, this SVD score was associated with poor outcomes in patients with AIS who received EVT (Arba et al., 2019). However, CT scan was less sensitive and accurate than MRI scan in detecting these markers. To date, there has been few studies using SVD scores based on MRI scans to evaluate the influence of SVD on the outcomes of patients receiving EVT.

Therefore, in this study, we assessed the SVD score based on MRI scan on admission before EVT and aimed to investigate the effect of SVD score on the clinical outcome of patients with AIS receiving EVT in a prospective nationwide registry.

## Materials and Methods

### Study Design and Subjects

As reported previously, ANGEL-ACT (Endovascular Treatment Key Technique and Emergency Work Flow Improvement of Acute Ischemic Stroke) was a prospective nationwide registry of patients with AIS with large vessel occlusion and treated with EVT at 111 hospitals from 26 provinces in China between November 2017 and March 2019 (Jia et al., 2021; Tong et al., 2021). The inclusion criteria of the registry were as follows: (1) age ≥ 18 years; (2) diagnosis of AIS caused by imaging-confirmed intracranial LVO, including internal carotid artery, middle cerebral artery (M1/M2), anterior cerebral artery (A1/A2), vertebrobasilar artery and posterior cerebral artery (P1); and (3) initiation of any type of EVT, including mechanical thrombectomy, intra-arterial thrombolysis, balloon angioplasty and stenting. The exclusion criteria were as follows: (1) presence of isolated cervical internal carotid artery or vertebral artery occlusion; and (2) no evidence of LVO on angiogram. The protocol was approved by the Ethics Committees of the Beijing Tiantan Hospital and all participating centers. Written informed consent was obtained from all the patients or their representatives. The unique identifier of the registry was NCT03370939 (Registration-URL^1^).

Patients in the ANGEL-ACT registry were included in the analysis according to the following criteria: (1) undergoing brain 1.5T or 3T MRI, including T1-weighted image, T2-weighted image, fluid-attenuated inversion recovery (FLAIR) image and diffusion-weighted imaging, on admission before EVT; and (2) with intact baseline, procedural and follow-up information. Those with inadequate MRI sequences or with poor image quality for SVD assessment were excluded from the current analysis.

### Data Collection

Data were derived from the ANGEL-ACT registry. Baseline information included demographic characteristics, medical history, vital signs, National Institutes of Health Stroke Scale (NIHSS) and laboratory and imaging results. Procedural information included time-metric data, use of IVT, EVT details and periprocedural management. Follow-up information included the following: (1) periprocedural complications; (2) NIHSS at 24 h and 7 days after the procedure; (3) functional outcome as measured by the modified Rankin Scale (mRS); and (4) adverse events within 90 days after the procedure.

### Small Vessel Disease Assessment

All MRI images were collected from participating centers in digital format and assessed centrally and independently by two trained readers who were blinded to the patients’ information. Disagreements were resolved by discussion and when necessary, a third reader with expertise in the field was consulted. To enhance generalizability of our results and in contrast to previous use of an SVD burden scale including microbleeds or perivascular spaces (Klarenbeek et al., 2013), the SVD scale in our study focused on the SVD features that were also detectable with CT scan (Arba et al., 2017b,^2019^). We applied a scale incorporating three markers of SVD, including lacune, white matter hyperintensities (WMH), and brain atrophy, to assess the burden of SVD.

According to the Standards for Reporting Vascular Changes on Neuroimaging (STRIVE) criteria (Wardlaw et al., 2013b), a lacune was defined as a round subcortical lesion with a signal similar to that produced by cerebrospinal fluid (CSF) and having a diameter between 3 and 15 mm. White matter hyperintensities were defined as hyperintense on T2-weighted and FLAIR images and isointense or hypointense on T1-weighted images. White matter hyperintensities were graded by the Van Swieten Scale (VSS) in the anterior and posterior region, yielding a 5-point ordinal scale (0–4) (van Swieten et al., 1990). We defined brain atrophy as cortical and deep, and the cortical or deep atrophy severity was rated as none, mild-moderate and severe against a reference MR brain template, yielding a 5-point ordinal scale (0–4) (IST-3 collaborative group, 2015; Arba et al., 2017b). Considering the possible effect of infarct, we measured SVD based on the multi-modal MRI images, including T1, T2, FLAIR, and DWI. The WMH burden was measured mainly on the contralateral hemisphere of acute anterior stroke. Furthermore, we also considered the stroke hemisphere to deal with the significant asymmetrical lesions. If the lesion emerged both on FLAIR and DWI images, we would subtract the part demonstrating high signals on DWI images, measure the remaining part on FLAIR images and compare with the WMH on the contralateral hemisphere.

In the SVD burden scale, the presence of each of the markers was assigned 1 point for each of the following: (1) severe white matter changes, with a VSS score ≥ 3; (2) number of lacunes ≥ 2; and (3) severe brain atrophy, with an atrophy score ≥ 3. Therefore, a 4-point ordinal score (0–3) was created and a higher score represented a higher burden of SVD. According to the SVD score, we categorized patients into two groups: the none-mild SVD burden group (SVD score = 0 or 1) and the moderate-severe SVD burden group (SVD score = 2 or 3).

### Outcome Measurement

The primary outcome was the mRS score at 90 days. The secondary outcomes included the following: (1) the proportions of mRS scores of 0–1, 0–2, and 0–3 at 90 days; (2) successful recanalization at the final angiogram, defined by the Thrombolysis in Cerebral Infarction [TICI] 2b–3; (3) changes in the NIHSS score 24 h after the procedure; and (4) changes in NIHSS score 7 days after the procedure. The safety outcomes were as follows: (1) death within 90 days; (2) any ICH within 24 h; and (3) symptomatic ICH within 24 h according to the Heidelberg Bleeding Classification (von Kummer et al., 2015). The 90-day outcomes were obtained via telephone interview by trained investigators blinded to the baseline information.

### Statistical Analysis

Continuous and ordinal variables were presented as medians (interquartile range [IQR]) and categorical variables were presented as numbers (percentages). For the comparison between groups, Mann–Whitney *U* tests were used for continuous and ordinal variables and χ^2^ tests or Fisher’s exact tests were used for categorical variables. For comparison of the outcomes between groups, the odds ratios (OR), common OR and β coefficients with 95% confidence intervals (CI) were analyzed by the binary or ordinal logistic regression model or generalized linear model according to the situation. In addition, we also used multivariable models to compare the outcomes between groups, adjusting for confounders including age, sex and NIHSS for their established relations with outcomes, and other baseline and procedural variables, with a significant difference of *P* < 0.1. We also did sensitivity analysis to illustrate the influence of the measurement method of WMH and SVD score on the outcomes. Finally, to investigate the associations of SVD features with the outcome, we applied an ordinal logistic regression model with mRS score at 90 days as the dependent variable and SVD features as independent variables and adjusted the analysis for the confounders mentioned above in a multivariable model. We considered a two-sided *P* < 0.05 as statistically significant and the statistical analysis was carried out by the SAS software, version 9.4 (SAS Institute, Inc, Cary, NC, United States).

## Results

**FIGURE 1:**
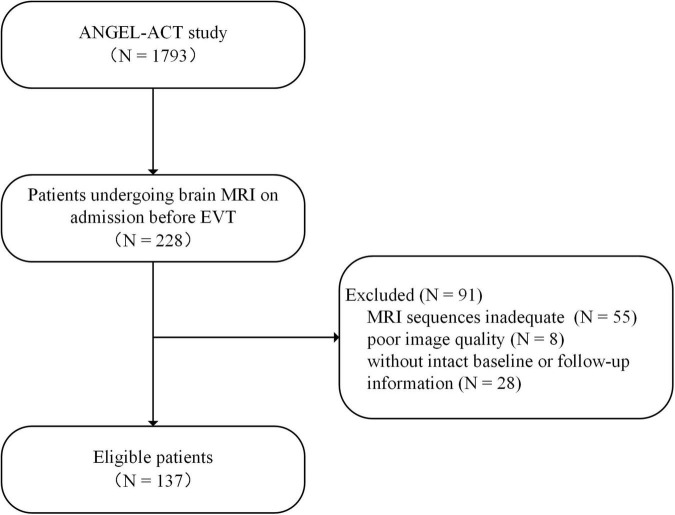
Flowchart of patient selection.

Among 1793 consecutive patients in the registry, 228 underwent brain MRI examination on admission before EVT. After excluding 55 patients with inadequate MRI sequences, 8 patients with poor image quality and 28 patients with missing baseline or follow-up information, a total of 137 patients were finally included in this analysis (Figure 1). Among all the patients, 71 (51.8%) were assessed with an SVD score of 0, 35 (25.5%) with an SVD score of 1, 22 (16.1%) with an SVD score of 2 and 9 (6.6%) with an SVD score of 3. The baseline and procedural characteristics of patients with different SVD scores were shown in Supplementary Table 1. Then, based on the SVD score, the entire patient sample was divided into two groups: the none-mild SVD burden group (*n* = 106, 77.4%) and the moderate-severe SVD burden group (*n* = 31, 22.6%).

### Baseline and Procedural Characteristics

As shown in Table 1, the baseline and procedural characteristics were compared between groups with different SVD scores and SVD burdens. Patients with moderate-severe SVD burden had a higher age (median, 67 versus 60, *P* = 0.04) and a higher number of stent retriever thrombectomy used during the whole procedure (median, 1 versus 1, *P* = 0.004) than those with none-mild SVD burden. Additionally, for the SVD features, patients with moderate-severe SVD burden had a larger score of WMH (median, 4 versus 1), a higher proportion of severe WMH (96.8% versus 13.2%), a larger number of lacune (median, 2 versus 0), a higher proportion of two or more lacunes (77.4% versus 11.3%), a larger score of brain atrophy (median, total: 3 versus 0, cortical: 1 versus 0, deep: 1 versus 0) and a higher proportion of severe brain atrophy (54.8% versus 8.5%) than those with none-mild SVD burden (all *P* < 0.001). Other baseline and procedural characteristics between the two groups were well-balanced.

**TABLE 1:** Baseline and procedural characteristics of patients with different SVD burdens.

Baseline and procedural variable	None-mild SVD burden (*N* = 106)	Moderate-severe SVD burden (*N* = 31)	*P*-value
Male	70 (66.0)	26 (83.9)	0.06
Age; median (IQR)	60 (53–67)	67 (56–73)	0.04
History of hypertension	63 (59.4)	22 (71.0)	0.24
History of diabetes	21 (19.8)	9 (29.0)	0.28
History of dyslipidemia	15 (14.2)	6 (19.4)	0.57
History of coronary heart disease	7 (6.6)	2 (6.4)	1.00
History of atrial fibrillation	27 (25.5)	6 (19.4)	0.48
Prior ischemic stroke	28 (26.4)	12 (38.7)	0.18
**Cigarette smoking**			
Never smoker	50 (47.2)	17 (54.8)	0.52
Ex-smoker	46 (43.4)	10 (32.3)	
Current smoker	10 (9.4)	4 (12.9)	
Systolic blood pressure, mmHg; median (IQR)	150 (130–165)	148 (138–160)	0.58
Diastolic blood pressure, mmHg; median (IQR)	85 (78–94)	90 (80–100)	0.27
**Premorbid mRS, median (IQR)**	0 (0–0)	0 (0–0)	0.06
NIHSS score before EVT, median (IQR)	13 (9–19)	15 (11–19)	0.18
ASPECTS, median (IQR)	7 (6–8)	7 (6–7)	0.18
**ASPCETS in anterior circulation**	7 (6–8)	7 (5–8)	0.21
**pc-ASPECTS in posterior circulation**	7 (5–8)	7 (6–7)	0.80
**SVD features**			
VSS score of WMH, median (IQR)	1 (1–2)	4 (3–4)	<0.001
Severe WMH	14 (13.2)	30 (96.8)	<0.001
Lacune number, median (IQR)	0 (0–1)	2 (2–4)	<0.001
Two or more lacunes	12 (11.3)	24 (77.4)	<0.001
Cortical brain atrophy score, median (IQR)	0 (0–1)	1 (1–1)	<0.001
Deep brain atrophy score, median (IQR)	0 (0–1)	1 (1–2)	<0.001
Total brain atrophy score, median (IQR)	0 (0–2)	3 (1–3)	<0.001
Severe brain atrophy	9 (8.5)	17 (54.8)	<0.001
**Occlusion site**			
Internal carotid artery	24 (22.6)	2 (6.45)	0.21
Middle cerebral artery M1 segment	37 (34.9)	12 (38.7)	
Vertebrobasilar artery	32 (30.2)	11 (35.5)	
Other intracranial arteries	13 (12.3)	6 (19.4)	
**Stroke subtype by TOAST criteria**			
Large artery atherosclerosis	66 (62.3)	23 (74.2)	0.37
Cardioembolism	30 (28.3)	7 (22.6)	
Other or unknown etiology	10 (9.4)	1 (3.2)	
Prior use of antiplatelet agents	16 (15.1)	6 (19.4)	0.58
Prior use of anticoagulants	5 (4.7)	1 (3.2)	1.00
Prior use of rt-PA	26 (24.5)	3 (9.7)	0.08
Use of heparin during the procedure	33 (31.1)	9 (29.0)	0.82
Use of GP2b3a inhibitor during the procedure	69 (65.1)	18 (58.1)	0.47
**No. of EVT modalities used during whole procedure**			
Stent retriever thrombectomy, median (IQR)	1 (1–2)	1 (0–1)	0.004
Aspiration thrombectomy, median (IQR)	0 (0–1)	0 (0–1)	0.82
IA thrombolysis, median (IQR)	0 (0–0)	0 (0–0)	0.73
Angioplasty, median (IQR)	0 (0–1)	0 (0–1)	0.20
Stenting, median (IQR)	0 (0–1)	0 (0–1)	0.78
Onset-to-puncture time, min; median (IQR)	387.5 (387.5–570)	462 (345–720)	0.13
**Onset-to-needle time for patients receiving rt-PA, min; median (IQR)**	367.5 (260–440)	382 (306–551)	0.75
Onset-to-recanalization time, min; median (IQR)	488 (404–675)	573 (462–897)	0.07

*ASPECTS, Alberta Stroke Program Early CT Score; pc-ASPECTS, Alberta Stroke Program Early CT Score of the posterior circulation; EVT, endovascular treatment; GP2b3a, Glycoproteins 2b and 3a; IQR, interquartile range; IA, intra-arterial; NIHSS, National Institute of Health stroke scale; rt-PA, recombinant tissue plasminogen activator; SVD, small vessel disease; TOAST, Trial of Org 10172 in Acute Stroke Treatment; VSS, Van Swieten Scale; WMH, white matter hyperintensities; Values are numbers with percentages in parentheses, unless indicated otherwise.*

### Outcome Measures

**FIGURE 2:**
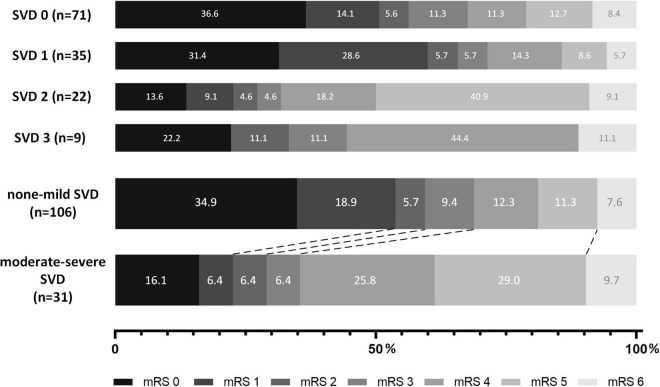
Shift of 90-day modified Rankin Scale (mRS) score of patients with different SVD scores and burdens.

As shown in Table 2, we used both univariable and multivariable models (adjusting for age, sex, NIHSS, onset-to-recanalization time, prior use of rt-PA and number of stent retriever thrombectomy during the procedure) to compare the outcomes between groups. Patients with moderate-severe SVD burden showed a significantly higher mRS score at 90 days than those with none-mild SVD burden (median, 4 versus 1 points, common OR 0.34, adjusted common OR 0.32 [95% CI, 0.14–0.69], *P* = 0.004). The shift on the mRS score at 90 days in the groups with different SVD scores and SVD burdens is shown in Figure 2. For the secondary outcomes, patients with moderate-severe SVD burden showed a significantly lower proportion of mRS 0–1 (22.6% versus 53.8%, common OR 0.25, adjusted common OR 0.25 [95% CI, 0.09–0.70], *P* = 0.008), mRS 0–2 (29.0% versus 59.4%, common OR 0.28, adjusted common OR 0.28 [95% CI, 0.11–0.74], *P* = 0.01), and mRS 0–3 (35.5% versus 68.9%, common OR 0.25, adjusted common OR 0.27 [95% CI, 0.10–0.73], *P* = 0.009) at 90 days. In addition, patients with moderate-severe SVD burden had a significantly smaller improvement of NIHSS at 24 h (median, –3 versus –3 points, β coefficient 2.16, adjusted β coefficient 4.02 [95% CI, 0.57, 7.48], *P* = 0.02) and 7 days (median, –4 versus –6 points, β coefficient 2.77, adjusted β coefficient 4.71 [95% CI, 1.06, 8.36], *P* = 0.01). There were no significant differences in successful recanalization at the final angiogram between two groups (*P* > 0.05). For the safety outcome, there were no significant differences between two groups in death within 90 days, any ICH within 24 h and symptomatic ICH within 24 h (all *P* > 0.05). The comparison results between the two groups were similar when only adjusting for the significant different baseline factors, that is, age and number of stent retriever thrombectomy. For the primary outcome, the moderate-severe SVD burden group also showed a significantly higher mRS score at 90 days than none-mild SVD burden group (adjusted odds ratio 0.34 [95% CI, 0.16–0.71], *P* = 0.002). There was also no significant difference in safety outcomes.

**TABLE 2:** Outcome measures of patients with none-mild SVD burden vs. moderate-severe SVD burden.

Outcome variable	None-mild SVD burden	Moderate-severe SVD burden	Unadjusted analysis		Adjusted analysis^†^	
			Effect size (95% CI)	*P*-value	Effect size (95% CI)	*P*-value
**Primary outcome**						
mRS at 90 days, median (IQR)	1 (0–4)	4 (2–5)	0.34 (0.16, 0.69)	0.001	0.32 (0.14, 0.69)^‡^	0.004
**Secondary outcome**						
mRS 0–1 at 90 days	57 (53.8)	7 (22.6)	0.25 (0.10, 0.63)	0.003	0.25 (0.09, 0.70)^§^	0.008
mRS 0–2 at 90 days	63 (59.4)	9 (29.0)	0.28 (0.12, 0.66)	0.004	0.28 (0.11, 0.74)^§^	0.01
mRS 0–3 at 90 days	73 (68.9)	11 (35.5)	0.25 (0.11, 0.58)	0.001	0.27 (0.10, 0.73)^§^	0.009
Successful recanalization at the final angiogram	88 (83.0)	22 (71.0)	0.50 (0.20, 1.26)	0.14	0.62 (0.21, 1.80)^§^	0.38
Change in NIHSS score at 24 h, median (IQR)	–3 (–8 to 0)	–3 (–5 to 0)	2.16 (–1.62, 5.93)	0.26	4.02 (0.57, 7.48)^¶^	0.02
Change in NIHSS score at 7 days, median (IQR)	–6 (–10 to –1)	–4 (–7 to 0)	2.77 (–1.23, 6.77)	0.17	4.71 (1.06, 8.36)^¶^	0.01
**Safety outcomes**						
Death within 90 days	8 (7.6)	3 (9.7)	1.33 (0.33, 5.27)	0.70	1.33 (0.19, 9.07)^§^	0.77
Any ICH within 24 h	12 (11.3)	3 (9.7)	0.84 (0.22, 3.18)	0.80	0.66 (0.13, 3.45)^§^	0.62
Symptomatic ICH within 24 h	9 (8.5)	2 (6.4)	0.74 (0.15, 3.64)	0.71	0.60 (0.10, 3.68)^§^	0.58

*ICH, intracerebral hemorrhage; IQR, interquartile range; mRS, modified Rankin Scale; NIHSS, National Institute of Health stroke scale; SVD, small vessel disease.*

*^†^Analysis adjusted for age, sex, NIHSS, onset-to-recanalization time, prior use of rt-PA, number of stent retriever thrombectomy during the procedure.*

*^‡^The common OR values were calculated by the ordinal logistic regression model and indicated the odds of improvement of 1 point on the mRS at 90 days.*

*^§^ The OR values were calculated by the binary logistic regression model.*

*^¶^ The β coefficients were calculated by the generalized linear model.*

There were 43 patients (32 with none-mild SVD burden and 11 with moderate-severe SVD burden) with posterior circulation stroke and 94 patients (74 with none-mild SVD burden and 20 with moderate-severe SVD burden) with anterior circulation stroke. The subgroup analysis in patients with anterior circulation stroke showed the moderate-severe SVD burden group had a significantly higher mRS score at 90 days (median, 4 versus 1 points, unadjusted odds ratio 0.35 [95% CI, 0.14–0.85], *P* = 0.02), while there was only a trend of worse outcome in moderate-severe SVD burden group without statistical significance in patients with posterior circulation stroke (median, 5 versus 1 points, unadjusted odds ratio 0.32 [95% CI, 0.09–1.12], *P* = 0.08).

### Sensitivity Analysis

Fazekas score has been widely used in WMH measurement. In another SVD score system (Klarenbeek et al., 2013), one point is allocated to WMH if periventricular WMH = 3 or deep WMH ≥ 2 according to the Fazekas score. To illustrate the influence of the measurement method of WMH, we also used the Fazekas score and found 100 patients with none-mild SVD burden and 37 patients with moderate to severe SVD burden. The moderate-severe SVD burden group also showed a significantly higher modified Rankin Scale score at 90 d (median, 4 versus 2 points, unadjusted odds ratio 0.42 [95% CI, 0.21–0.82], *P* = 0.01; adjusted odds ratio 0.44 [95% CI, 0.21–0.91], *P* = 0.03). There was also no significant difference in death within 90 d and symptomatic intracranial hemorrhage within 24 h between two groups. Hence, although we applied another WMH method, the main outcomes were similar.

There were also a very small number of patients receiving SWI on admission before EVT in the registry. Among the 137 patients included in the study, we found that there were 73 patients with SWI. In the subgroup, we used another new SVD score (SVD score 2) (Klarenbeek et al., 2013), including WMH, lacune, PVS and microbleed, to evaluate the SVD burden and compared with the original SVD score (SVD score 1). In the subgroup, 56 patients were with the none-mild SVD burden and 17 with moderate-severe SVD burden according to the SVD score 1; 63 patients with none-mild SVD burden and 10 with moderate-severe SVD burden according to the SVD score 2. When applying SVD score 1, the moderate-severe SVD burden group showed a significantly higher mRS score at 90 days (median, 4 versus 2 points, unadjusted odds ratio 0.30 [95% CI, 0.11–0.82], *P* = 0.02; adjusted odds ratio 0.32 [95% CI, 0.11–0.89], *P* = 0.03, adjusted for age and sex). When applying SVD score 2, the moderate-severe SVD burden group showed a higher but not significant mRS score at 90 d with none-mild SVD burden group (median, 4 versus 3 points, unadjusted odds ratio 0.66 [95% CI, 0.20–2.14], *P* = 0.48; adjusted odds ratio 0.78 [95% CI, 0.23–2.59], *P* = 0.68, adjusted for age and sex). There was no significant difference in death within 90 d and symptomatic intracranial hemorrhage within 24 h when using either of the two scores.

### Small Vessel Disease Features and Outcome

As shown in Table 3, for the original scores of each SVD marker, the VSS score of WMH (common OR, 0.74 [95% CI, 0.59–0.92], *P* = 0.007), deep brain atrophy score (common OR, 0.66 [95% CI, 0.44–0.98], *P* = 0.04), and total brain atrophy score (common OR, 0.78 [95% CI, 0.61–0.99], *P* = 0.04) were significantly associated with the primary outcome (mRS score at 90 days) in the univariable logistic regression analysis, whereas the lacune number and cortical brain atrophy score were not. For the SVD burden scores of each SVD marker, severe brain atrophy (common OR, 0.40 [95% CI, 0.19–0.86], *P* = 0.02) was significantly associated with the primary outcome, whereas lacune number ≥ 2 and severe WMH were not. In addition, the total SVD burden score (common OR, 0.70 [95% CI, 0.51–0.96], *P* = 0.03) was also associated with the primary outcome.

**TABLE 3:** Ordinal logistic regression analysis with mRS score at 90 days as the dependent variable and SVD features as independent variables.

SVD features	Unadjusted analysis		Adjusted analysis^†^	
	Common OR (95% CI)*	*P*-value	Common OR (95% CI)*	*P*-value
**Original scores of each SVD marker**				
VSS score of WMH	0.74 (0.59, 0.92)	0.007	0.66 (0.51, 0.86)	0.002
Lacune number	0.95 (0.83, 1.10)	0.50	0.94 (0.81, 1.10)	0.46
Cortical brain atrophy score	0.65 (0.40, 1.07)	0.09	0.55 (0.30, 0.99)	0.04
Deep brain atrophy score	0.66 (0.44, 0.98)	0.04	0.54 (0.34, 0.87)	0.008
Total brain atrophy score	0.78 (0.61, 0.99)	0.04	0.67 (0.50, 0.90)	0.008
**SVD burden scores of each SVD marker**				
Severe WMH	0.54 (0.28, 1.01)	0.06	0.48 (0.23, 0.98)	0.04
Two or more lacunes	0.82 (0.42, 1.61)	0.56	0.84 (0.41, 1.73)	0.64
Severe brain atrophy	0.40 (0.19, 0.86)	0.02	0.31 (0.14, 0.72)	0.006
Total SVD burden score	0.70 (0.51, 0.96)	0.03	0.64 (0.41, 0.91)	0.01

*mRS, modified Rankin Scale; SVD, small vessel disease; VSS, Van Swieten Scale; WMH, white matter hyperintensities.*

*^†^Analysis adjusted for age, sex, NIHSS, onset-to-recanalization time, prior use of rt-PA, number of stent retriever thrombectomy during the procedure.*

*The common OR values indicated the odds of improvement of 1 point on the mRS at 90 days.

After adjusting for age, sex, NIHSS, onset-to-recanalization time, prior use of recombinant tissue plasminogen activator (rt-PA) and number of stent retriever thrombectomy during the procedure, these associations were still statistically significant for the VSS score of WMH (adjusted common OR, 0.66 [95% CI, 0.51–0.86], *P* = 0.002), deep brain atrophy score (adjusted common OR, 0.54 [95% CI, 0.34–0.87], *P* = 0.008), total brain atrophy score (adjusted common OR, 0.67 [95% CI, 0.50–0.90], *P* = 0.008), severe brain atrophy (adjusted common OR, 0.31 [95% CI, 0.14–0.72], *P* = 0.006) and the total SVD burden score (adjusted common OR, 0.64 [95% CI, 0.41–0.91], *P* = 0.01). In addition, the cortical brain atrophy score (adjusted common OR, 0.55 [95% CI, 0.30–0.99], *P* = 0.04) and severe WMH (adjusted common OR, 0.48 [95% CI, 0.23–0.98], *P* = 0.04) were also significantly associated with the primary outcome in the multivariable analysis, whereas the lacune number and lacune number ≥ 2 remained not.

## Discussion

In this study, we found that the SVD burden measured by the SVD score on the baseline MRI was associated with the functional outcomes in patients with acute ischemic stroke caused by large vessel occlusion who received EVT. Patients with moderate-severe SVD burden had worse early and late functional outcomes than those with none-mild SVD burden, but there was no difference in sICH and mortality between the two groups. Additionally, for each single SVD marker, brain atrophy and WMH were associated with functional outcomes, whereas lacunes were not, suggesting that brain atrophy and WMH were the major drivers in the relation between SVD scores and the outcome of patients after EVT. Our results suggested that the SVD score, as a surrogate marker of SVD reflecting the overall brain pathology, may act as a good predictor of outcomes in patients with AIS who receive EVT.

Outcomes in patients with SVD who receive revascularization treatment have mainly been investigated in the context of receiving IVT (Arba et al., 2017a; Liu et al., 2019), partly in EVT. One study using the same SVD score as our study and based on MRI scan showed that SVD negatively affected the outcomes in patients receiving IVT and among these SVD markers, only WMH was associated with the clinical outcome (Arba et al., 2017a). Another study applying a different MRI-based SVD score also identified the association of SVD with poor outcomes at 90 days in patients receiving IVT (Liu et al., 2019). These studies suggested the predictive value of the SVD score in ischemic stroke. Similar to the findings in studies of IVT, we also found that there was an unfavorable effect of SVD on the functional outcomes in patients with AIS who had received EVT. The results of our study, the first of its kind based on MRI, confirmed the association of SVD burden and outcomes demonstrated by a previous study based on the CT scan (Arba et al., 2019). For each single SVD marker, both the previous CT-based study and our MRI-based study did not show the association between lacunes and the outcomes. Although lacunes were found to be associated with the outcomes in patients receiving IVT (Huo et al., 2019), the independent association between lacunes and outcomes in patients receiving EVT has not been found so far. In addition, our results showed that not only brain atrophy as reported in the previous CT study, but also WMH was associated with the functional outcomes.

Brain atrophy is common in patients with stroke and is usually recognized as a risk factor for cognitive impairment (Fox and Schott, 2004). The second analysis of the third International Stroke Trial (IST-3) used a measurement method similar to the one we used to show that brain atrophy was associated with the reduction of independence in patients receiving IVT (IST-3 collaborative group, 2015). For patients receiving EVT, one study using an automated measurement of CSF volume to assess brain atrophy also showed that brain atrophy was associated with reduced functional independence (Diprose et al., 2019). Additionally, a recent study using the brain atrophy index also demonstrated that the brain atrophy was associated with the mortality in patients after EVT (Lauksio et al., 2021). Although the methods measuring brain atrophy differed, these studies revealed the association between brain atrophy and outcome in patients with stroke who received revascularization treatment. However, we must be cautious when interpreting the reasons underlying the associations found in these studies. Although brain atrophy has been recognized as a marker of SVD (Wardlaw et al., 2013b), brain atrophy is also common in neurodegenerative diseases (Fox and Schott, 2004).

White matter hyperintensities is also common in patients with stroke, and previous studies in patients with AIS who received IVT have shown that WMH was associated with less favorable functional outcomes and increased the risk of sICH (Charidimou et al., 2016; Kongbunkiat et al., 2017). For patients receiving EVT, the results have not been consistent in previous studies. Results from two retrospective studies showed no association between the functional outcome and WMH burden as assessed by WMH volume and Fazekas score (Atchaneeyasakul et al., 2017; Mechtouff et al., 2020). In contrast, two recent prospective multi-center studies with larger samples showed that patients with high WMH burden assessed by WMH volume and VSS score had a higher risk of poor functional outcome but a similar rate of sICH (Boulouis et al., 2019; Mistry et al., 2020). Overall, evidence is accumulating that WMH burden is associated with outcomes in patients with AIS who receive EVT and this association must be validated in further studies, particularly in randomized trials.

Microbleed is also a image marker of SVD. We used two kinds of SVD scores in the study: one score without assessment of microbleed applied in the whole population and another score with four markers including microbleed applied in the subgroup analysis. It’s noted that there was no significant difference in the safety outcomes when using either of the two scores. Previous studies also showed that the presence of CMBs was not significantly associated with the risk of ICH in patients receiving EVT (Shi et al., 2016; Derraz et al., 2021). However, considering the small sample of the study, we should be cautious when interpreting the relationship between SVD burden and the safety outcome.

The mechanisms underlying the association between SVD and functional outcomes have not been elucidated. The SVD score can reflect the severity of brain parenchymal lesions in SVD. A higher SVD score denotes the presence of a more severe brain lesion, which makes the brain more susceptible to stroke (Kim and Lee, 2015). SVD was also associated with cognitive impairment, which may also affect recovery and daily performance, leading to a lower mRS score (Jiang et al., 2019). In addition, advanced MRI techniques provided new possible clues for the mechanisms behind these phenomena. One perfusion-weighted imaging study showed a higher SVD burden was associated with increasing blood-brain barrier leakage in the ischemic area (Arba et al., 2017b), which may explain the increased sICH in patients receiving intravenous thrombolysis. Another study on diffusion tensor imaging has shown that the SVD burden was associated with decreased function of the white matter network, which can also influence brain recovery (Kim et al., 2015).

Our study had some limitations. First, not all SVD markers were included in the determination of SVD score, and therefore the impact of SVD on the outcome may not have been fully estimated. Because susceptibility weighted imaging (SWI) or gradient echo (GRE) sequences are not routinely applied in patients with AIS in most hospitals in China, an assessment of cerebral microbleeds was not available for most patients in the registry. The subgroup analysis using another SVD score with four SVD markers including microbleed also showed a trend of worse outcome in moderate-severe SVD burden group. The SVD score in our study only included the SVD markers that can be evaluated on routine MRI sequences, which enables translation of our results into real-world clinical practice. We acknowledge that the impact of SVD still needs further validation using different SVD scores that include more SVD markers. Second, there may have been a selection bias in our study. Because we used MRI as the evaluation tool, patients who were unable to receive an MRI scan or had poor image quality may have had more severe AIS but nonetheless would have been excluded from our study. However, the aim of our study was to evaluate SVD markers using a more accurate tool and so the selection bias could not be fully avoided. Third, the proportion of patients who were eligible for our study in the registry was small, thus our sample size was smaller compared to previous studies using CT scan or assessing only one SVD marker. The strengths of our study were its multi-institutional design and the blinded evaluation of SVD score based on routine MRI. Notably, our study was based on a registry from China and whether our results are applicable for other populations needs further validation. Forth, we had adjusted seven confounders in the multivariable model and the number was large considering the relative small sample size. As a supplementary analysis, we also provided the results only adjusting for two confounders and the conclusions were similar. Further studies, informed by our results, are needed to investigate the impact of SVD on the outcome of EVT through different SVD scores in a larger and more representative population.

## Conclusion

In conclusion, our results demonstrate that the SVD burden was negatively associated with the functional outcome in patients with AIS receiving EVT. Compared to those with none-mild SVD burden, patients with moderate-severe SVD burden had worse early and late functional outcomes but a similar sICH rate. Our results suggested the value of MRI-based assessment of SVD burden in predicting the functional outcome in patients with AIS who received EVT. Further studies to validate our findings in larger cohorts or randomized trials are warranted.

## Data Availability Statement

The original contributions presented in this study are included in the article/Supplementary Material, further inquiries can be directed to the corresponding author/s.

## Ethics Statement

The protocol was approved by the Ethics Committees of the Beijing Tiantan Hospital and all participating centers. Written informed consent was obtained from all the patients or their representatives. The patients/participants provided their written informed consent to participate in this study.

## Author Contributions

BJ, XTo, JZ, LW, XZ, YG, YZ, and WZ collected the data. XTi, HH, and AW processed the statistical data. HH drafted and revised the manuscript. YLW, YJW, and ZM designed the original ANGEL-ACT study. ZM and XL designed and guided this study. All authors read and approved the final manuscript.

## Conflict of Interest

The authors declare that the research was conducted in the absence of any commercial or financial relationships that could be construed as a potential conflict of interest.

## Publisher’s Note

All claims expressed in this article are solely those of the authors and do not necessarily represent those of their affiliated organizations, or those of the publisher, the editors and the reviewers. Any product that may be evaluated in this article, or claim that may be made by its manufacturer, is not guaranteed or endorsed by the publisher.

## Footnotes

1
http://www.clinicaltrials.gov
