# Supplementary material for: Effect of Cerebral Small Vessel Disease Burden on Outcomes in Patients With Acute Ischemic Stroke Receiving Endovascular Treatment
Source: Front Aging Neurosci. 2022 Jun 13;14:800617. doi: 10.3389/fnagi.2022.800617 (PMC9234259; doi:10.3389/fnagi.2022.800617)
Supplement: Supplementary file 1 [file Table_1.docx]

**Supplemental Table 1. Baseline and procedural characteristics of patients with different SVD scores**

| **Baseline and procedural variable** | **SVD 0**  **（N=71）** | **SVD 1**  **（N=35）** | **SVD 2 （N=22）** | **SVD 3**  **（N=9）** | ***P* value** |
| --- | --- | --- | --- | --- | --- |
| Male | 45 (63.4) | 25 (71.4) | 19 (86.4) | 7 (77.8) | 0.20 |
| Age; median (IQR) | 58 (50-66) | 67 (61-74) | 66 (53-72) | 67 (60-73) | <0.01 |
| History of hypertension | 39 (54.9) | 24 (68.6) | 16 (72.7) | 6 (66.7) | 0.35 |
| History of diabetes | 13 (18.3) | 8 (22.9) | 8 (36.4) | 1 (11.1) | 0.31 |
| History of dyslipidemia | 8 (11.3) | 7 (20.0) | 5 (22.7) | 1 (11.1) | 0.44 |
| History of coronary heart disease | 3 (4.2) | 4 (11.4) | 2 (9.1) | 0 (0) | 0.39 |
| History of atrial fibrillation | 15 (21.1) | 12 (.34.3) | 5 (22.7) | 1 (11.1) | 0.36 |
| Prior ischemic stroke | 13 (18.3) | 15 (42.9) | 8 (36.4) | 4 (44.4) | 0.03 |
| Cigarette smoking |  |  |  |  |  |
| Never smoker | 34 (47.9) | 16 (45.7) | 12 (54.6) | 5 (55.6) | 0.78 |
| Ex-smoker | 32 (45.1) | 14 (40.0) | 7 (31..8) | 3 (33.3) |  |
| Current smoker | 5 (7.0) | 5 (14.3) | 3 (13.6) | 1 (11.1) |  |
| Systolic blood pressure, mmHg; median (IQR) | 150 (130-160) | 153 (140-175) | 142.5 (120-164) | 150 (146-160) | 0.34 |
| Diastolic blood pressure, mmHg; median (IQR) | 85 (79-90) | 87 (76-97) | 89 (80-95) | 90 (80-100) | 0.62 |
| NIHSS score, median (IQR) | 12 (9-18) | 14 (9-19) | 16 (13-26) | 15 (8-18) | 0.39 |
| ASPECTS, median (IQR) | 7 (5-9) | 7 (6-8) | 7 (5-7) | 7 (6-8) | 0.50 |
| SVD features |  |  |  |  |  |
| VSS score of WMH, median (IQR) | 1 (0-2) | 2 (2-3) | 4 (3-4) | 4 (4-4) | <0.01 |
| Severe WMH | 0 (0) | 14 (40.0) | 21 (95.4) | 9 (100.0) | <0.01 |
| Lacune number, median (IQR) | 0 (0-0) | 1 (0-2) | 2 (1-4) | 2 (2-5) | <0.01 |
| Two or more lacunes | 0 (0) | 12 (34.3) | 15 (68.2) | 9 (100.0) | <0.01 |
| Cortical brain atrophy score, median (IQR) | 0 (0-0) | 1 (0-1) | 1 (0-1) | 1 (1-1) | <0.01 |
| Deep brain atrophy score, median (IQR) | 0 (0-0) | 1 (0-2) | 1 (0-1) | 2 (2-2) | <0.01 |
| Total brain atrophy score, median (IQR) | 0 (0-0) | 2 (1-3) | 2 (1-3) | 3 (3-3) | <0.01 |
| Severe brain atrophy | 0 (0) | 9 (25.7) | 8 (36.4) | 9 (100.0) | <0.01 |
| Occlusion site |  |  |  |  |  |
| Internal carotid artery | 19 (26.8) | 5 (14.3) | 1 (4.5) | 1 (11.1) | 0.07 |
| Middle cerebral artery M1 segment | 24 (33.8) | 13 (37.1) | 8 (36.4) | 4 (44.4) |  |
| Vertebrobasilar artery | 17 (23.9) | 15 (42.9) | 10 (45.4) | 1 (11.1) |  |
| Other intracranial arteries | 11 (15.5) | 2 (5.7) | 3 (13.6) | 3 (33.3) |  |
| Stroke subtype by TOAST criteria |  |  |  |  |  |
| Large artery atherosclerosis | 46 (64.8) | 20 (57.1) | 15 (68.2) | 8 (88.9) | 0.60 |
| Cardioembolism | 17 (23.9) | 13 (37.1) | 6 (27.3) | 1 (11.1) |  |
| Other or unknown etiology | 8 (11.3) | 2 (5.7) | 1 (4.6) | 0 (0) |  |
| Prior use of antiplatelet agents | 6 (8.4) | 10 (28.6) | 4 (18.2) | 2 (22.2) | 0.04 |
| Prior use of anticoagulants | 3 (4.2) | 2 (5.7) | 1 (4.6) | 0 (0) | 1.00 |
| Prior use of rt-PA | 19 (26.8) | 7 (20.0) | 2 (9.1) | 1 (11.1) | 0.33 |
| Use of heparin during the procedure | 22 (31.0) | 11 (31.4) | 8 (36.4) | 1 (11.1) | 0.58 |
| Use of GP2b3a inhibitor during the procedure | 47 (66.2) | 22 (62.9) | 15 (68.2) | 3 (33.3) | 0.26 |
| No. of EVT modalities used during the whole procedure |  |  |  |  |  |
| Stent retriever thrombectomy, median (IQR) | 1 (1-2) | 1 (1-3) | 1 (1-2) | 0 (0-0) | 0.02 |
| Aspiration thrombectomy, median (IQR) | 0 (0-1) | 0 (0-1) | 0 (0-1) | 0 (0-1) | 0.73 |
| IA thrombolysis, median (IQR) | 0 (0-0) | 0 (0-0) | 0 (0-0) | 0 (0-0) | 0.28 |
| Angioplasty, median (IQR) | 0 (0-1) | 0 (0-1) | 0 (0-1) | 0 (0-2) | 0.60 |
| Stenting, median (IQR) | 0 (0-1) | 0 (0-1) | 0 (0-1) | 0 (0-0) | 0.61 |
| Onset-to-puncture time, min; median (IQR) | 395 (305-618) | 360 (255-510) | 389 (285-620) | 585 (505-895) | 0.06 |
| Onset-to-recanalization time, min; median (IQR) | 501 (406-749) | 468 (357-664) | 539 (449-695) | 750 (575-949) | 0.07 |

ASPECTS, Alberta Stroke Program Early CT Score; GP2b3a, Glycoproteins 2b and 3a; IQR, interquartile range; IA, intra-artery; TOAST, Trial of Org 10172 in Acute Stroke Treatment. Values are numbers with percentages in parentheses, unless indicated otherwise.

List of ANGEL-ACT study group

Beijing Tiantan Hospital, Beijing, China: Zhongrong Miao, MD; Langfang Changzheng Hospital, Hebei, China: Liqiang Gui, MD; Liaocheng Third People’s Hospital, Shandong, China: Cunfeng Song, MD; The First People’s Hospital of Changzhou, Jiangsu, China: Ya Peng, MD; The Second Affiliated Hospital of Nanjing Medical University, Jiangsu, China: Jin Wu, MD; Fengrun District People’s Hospital of Tangshan City, Hebei , China: Shijun Zhao, MD; SiPing Central People’s Hospital, Jilin, China: Junfeng Zhao, MD; Yijishan Hospital of Wannan Medical College, Anhui, China: Zhiming Zhou, MD; The 2nd Affiliated Hospital of Harbin Medical University, Heilongjiang, China: Yongli Li, MD; The Central Hospital of Wuhan, Hubei, China: Ping Jing, MD; The First Hospital of Shijiazhuang, Hebei, China: Lei Yang, MD; Shenzhen Hospital of Southern Medical University, Guangdong, China: Yajie Liu, MD; The People’s Hospital of Longhua, Guangdong, China: Qingshi Zhao, MD; Jingjiang People’s Hospital, Jiangsu, China: Yan Liu, MD; The Third People’s Hospital of Hubei Province, Hubei, China: Xiaoxiang Peng, MD; The Second Affiliated Hospital of Guangzhou Medical University, Guangdong, China: Qingchun Gao, MD; Tianjin TEDA Hospital, Tianjin, China: Zaiyu Guo, MD; Zhangzhou Affiliated Hospital of Fujian Medical University, Fujian, China: Wenhuo Chen, MD; Taiyuan Central Hospital, Shanxi, China: Weirong Li, MD; The First Affiliated Hospital of Xinjiang Medical University, Xinjiang, China: Xiaojiang Cheng, MD; Affiliated Drum Tower Hospital of Nanjing University Medical School, Jiangsu, China: Yun Xu, MD; The First People’s Hospital of Wenling, Zhejiang, China: Yongqiang Zhang, MD; The Second Affiliated Hospital of Xi’an Jiaotong University, Shaanxi , China: Guilian Zhang, MD; The First People’s Hospital of Yulin, Guangxi, China: Yijiu Lu, MD; Zhenjiang First People’s Hospital, Jiangsu, China: Xinyu Lu, MD;
Qitaihe Coal General Hospital Heilongjiang, China: Dengxiang Wang, MD; People’s Hospital of Tangshan City, Hebei , China: Yan Wang, MD; Affiliated Hospital of Guilin Medical University, Guangxi, China: Hao Li, MD; The Affiliated Hospital of Guizhou Medical University, Guizhou Province, China: Yang Hua, MD; The Affiliated Hospital of Xuzhou Medical University, Jiangsu, China: Deqin Geng, MD; Qingdao Central Hospital, ShanDong, China: Haicheng Yuan, MD; The Fourth People’s Hospital of Langfang City, Hebei , China: Hongwei Wang, MD; Beijing Daxing hospital, Beijing, China: Haihua Yang, MD; Weifang People’s Hospital,ShanDong, China: Zengwu Wang, MD; Luoyang General Hospital Affiliated to Zhengzhou University, Henan, China: Liping Wei, MD; Dongguan Kanghua Hospital, Guangdong, China: Xuancong Liufu, MD; Shunde Hospital of Southern Medical University, Guangdong, China: Xiangqun Shi, MD; Handan Central Hospital, Hebei, China: Juntao Li, MD; The 981 hospital of the Chinese People’s Liberation Army, Hebei , China: Wenwu Yang, MD; Linfen people’s Hospital, Shanxi, China: Wenji Jing, MD; Anshun people’s Hospital of Guizhou , China: Xiang Yong, MD; Changle People’s Hospital, Shandong, China: Leyuan Wang, MD; The Second People’s Hospital of Dongying, Shandong, China: Chunlei Li, MD; Tangshan Gongren hospital, HeBei , China: Yibin Cao, MD; PLA 985th Hospital of the Joint Logistics Support Force, Shanxi, China: Qingfeng Zhu, MD; Gaomi People’s Hospital, ShanDong, China: Peng Zhang, MD; Tongji Hospital, Tongji Medical College, Huazhong University of Science and Technology, Hubei, China: Xiang Luo, MD; Chongqing Sanxia Center Hospital, Chongqing, China: Shengli Chen, MD; Hospital of Traditional Chinese Medicine of Qiannan, Guizhou , China: WenWu Peng, MD; Guangdong Hospital of Chinese Medicine, Guangdong, China: Lixin Wang, MD; People’s hospital of Yangjiang，Guangdong, China: Xue Wen, MD; The Third Affiliated Hospital of CQMU, Chongqing, China: Shugui Shi, MD; General Hospital of The Yangtze River Shipping, Hubei, China: Wanming Wang,
MD; First People’s Hospital of Bijie City,Guizhou, China: Wang Bo, MD; Suqian People’s Hospital of Nanjing Drum-Tower Hospital Group, Jiangsu, China: Pu Yuan, MD; Weifang TCM Hospital, Shandong, China: Dong Wang, MD; The Third Affiliated Hospital of Guangzhou Medical University, Guangdong, China: Haitao Guan, MD; Karamay Central hospital, Xinjiang, China: Wenbao Liang, MD; The third people’s Hospital of Xinjiang Uygur Autonomous Region, Xinjiang, China: Daliang Ma, MD; Wulanchabu City Central Hospital, Inner Mongolia, China: Long Chen, MD; Hospital of Xinjiang Production & Construction Corps , Xinjiang, China: Yan Xiao, MD; Jiaozuo Second people’s hospital, Henan, China: Xiangdong Xie, MD; 904th Hospital of Joint Logistic Support Force of PLA, Jiangsu, China: Zhonghua Shi, MD; Ganzhou People’s Hospital, Jiangxi, China: Xiangjun Zeng, MD; 967 Hospital of the Joint Logistics Support Force of PLA, Liaoning, China: Fanfan Su, MD; The Affiliated Hospital of Northwest University Xi’an No.3 Hospital, Shaanxi , China: MingZe Chang, MD; The Second Hospital of Liao Cheng, Shandong, China: Jijun Yin, MD; Jilin Province People’s Hospital, Jilin, China: Hongxia Sun, MD;
People’s Hospital of Huanghua City, Hebei, China: Chong Li, MD; Shanghai Forth People’s Hospital, Shanghai, China: Yong Bi, MD; Wanbei Coal-electricity Group Gneral Hospital, Anhui, China: Gang Xie, MD; Shanghai Jiao Tong University Affiliated Sixth People’s Hospital, Shanghai, China: Yuwu Zhao, MD; Binzhou Medical University Hospital, Shandong, China: Chao Wang, MD; The 988 hospital of the people’s liberation army, Henan , China: Peng Zhang, MD; Linyi People’s Hospital, Shandong, China: Xianjun Wang, MD; Yingkou City Central Hospital, Liaoning, China: Dongqun Li, MD; Yantaishan Hospital, Shandong, China: Hui Liang, MD; Mianyang Central hospital, Sichuan, China: Zhonglun Chen, MD; Chengdu Fifth People’s Hospital, Sichuan, China: Yan Wang, MD; Hengshui Fifth Hospital of Heng shui City, HeBei, China: Yu Xin, Wang, MD; Second Hospital of Dalian Medical University, Liaoning, China: Lin Yin, MD; Boai Hospital of Zhongshan, Guangdong, China: HongKai Qiu, MD; The First People’s
Hospital of Yibin, Sichuan , China: Jun Wei, MD; Shanxi provincial people’s hospital, Shanxi, China: Yaxuan Sun, MD; Shandong Provincial Third Hospital, Cheeloo College of Medicine, Shandong University, Shandong, China: Xiaoya Feng, MD; Chuxiong State People’s Hospital, Chuxiong, Yunnan, China: Weihua Wu, MD; The Fourth Affiliated Hospital of China Medical University, Liaoning, China: Lianbo Gao, MD; Taihe Hospital, Shiyan, Hubei , China: Zhibing Ai, MD; Qingdao Municipal Hospital，Shandong, China: Tan Lan, MD; The First People’s Hospital of Yunnan Province, Yunnan, China: Li Ding, MD; The NO.2 People’s Hospital of Lanzhou. Gansu, China: Qilong Liang, MD; Taizhou First People’s Hospital, Zhejiang, China: Zhimin Wang, MD; Hunan Provincial People’s Hospital, Hunan, China: Jianwen Yang, MD; First People’s Hospital of Changde City, Hunan, China: Ping Xu, MD; Zhejiang Yuyao People’s Hospital, Zhejiang, China: Wei Dong, MD; AideBao Hospital, HeBei , China: Quanle Zheng, MD; The First Hospital of Fangshan District, Beijing, China: Zhenyun Zhu, MD; Tianjin Xiqing Hospital, Tianjin,China: Liyue Zhao, MD; People’s Hospital of Zunhua, Hebei, China: Qingbo Meng, MD; Xingtai Third
Hospital, Hebei, China: Yuqing Wei, MD; Qingyuan People’s Hospital, Guangdong , China: Xianglin Chen, MD; Fengcheng City Central Hospital，Liaoning, China: Wei Wang, MD; People’s Hospital of Hejian City, Hebei , China: Dong Sun, MD; Hangzhou Third People’s Hospital, Zhejiang, China: Yongxing Yan, MD; Xiangtan Central Hospital, Hunan, China: Guangxiong Yuan, MD; People’s Hospital of Nanpi Country, Hebei , China: Yadong Yang, MD; Liuzhou Railway Central Hospital, Guangxi, China: Jianfeng Zhou, MD; Maoming People’s Hospital, Guangdong, China: Zhi Yang, MD; Tongde Hospital of Zhejiang Province, Zhejiang, China: Zhenzhong Zhang, MD; The First Affiliated Hospital of Jinzhou Medical University, Liaoning, China: Ning Guan, MD; Xishan coal electricity group worker general hospital, Shaanxi, China: Huihong Wang, MD
